# Influence des comorbidités sur l’évolution et le pronostic des patients porteurs de broncho-pneumopathie chronique obstructive dans un centre tunisien

**DOI:** 10.11604/pamj.2020.36.76.21511

**Published:** 2020-06-09

**Authors:** Ahmed Ben Saad, Lobna Loued, Samah Joobeur, Asma Migaou, Saousen Cheikh Mhamed, Naceur Rouatbi, Nesrine Fahem

**Affiliations:** 1 Service de Pneumologie et d’Allergologie, Hôpital Universitaire Fattouma Bourguiba, Rue 1er juin, Monastir, Tunisie

**Keywords:** Broncho-pneumopathie chronique obstructive, comorbidités, prévalence, évolution, pronostic, Chronic obstructive pulmonary disease, co-morbidity, prevalence, evolution, prognosis

## Abstract

**Introduction:**

la broncho-pneumopathie chronique obstructive (BPCO) s’associe fréquemment avec des comorbidités. L’objectif de notre travail est d'étudier l'impact des comorbidités sur l’évolution et le pronostic de la BPCO.

**Méthodes:**

il s’agit d’une étude rétrospective incluant des patients porteurs de BPCO hospitalisés et/ou suivis à la consultation de Pneumologie au Centre Hospitalo-Universitaire Fattouma Bourguiba de Monastir entre Janvier 2000 jusqu’à Décembre 2017. Les patients ont été répartis initialement en deux groupes, le groupe G0: BPCO isolée et le groupe G1: au moins une comorbidité. Nous avons divisé les patients du groupe G1 en deux sous-groupes: Groupe A: patients ayant 1-2 comorbidités et Groupe B: ≥ 3 comorbidités associées. Nous avons comparé les différents paramètres de sévérité de la BPCO entre les différents groupes.

**Résultats:**

en tout 1152 patients BPCO ont été inclus. Soixante-dix-neuf pourcent des patients avaient au moins une pathologie chronique associée à leur BPCO. La présence d’au moins une comorbidité était associée à l'augmentation du nombre des exacerbations sévères (p = 0,004), avec plus de recours à l’oxygène longue durée (p = 0,006) et à une survie réduite (p = 0,001). De même, un nombre de comorbidités plus important (≥ 3 comorbidités) était associé à une inflammation systémique plus importante, à un recours plus fréquent à la ventilation mécanique ou la ventilation non invasive (p = 0,04) et à une survie réduite (p = 0,05).

**Conclusion:**

la présence de comorbidités au cours de la BPCO s’associe à une sévérité plus importante et un pronostic plus sombre de la maladie.

Abstract

## Abstract (en)

**Introduction:**

chronic obstructive pulmonary disease (COPD) has been frequently associated with co-morbidities. The purpose of our study is to analyze the impact of co-morbidities on the progression and prognosis of COPD.

**Methods:**

we conducted a retrospective study of patients with COPD hospitalized and/or followed up in the Department of Pneumology at the Fattouma Bourguiba University Hospital in Monastir over the period January 2000-December 2017. The patients were initially divided into two groups, the G0 group (isolated COPD) and the G1 group (with at least one comorbidity). Patients in the G1 group were divided in two subgroups: A group (patients with 1-2 co-morbidities) and B group (≥ 3 comorbidities associated). Different parameters of COPD severity were compared between the different groups.

**Results:**

a total of 1152 patients with COPD were enrolled. Seventy-nine percent of patients had at least a chronic disease associated with COPD. The presence of at least one co-morbidity was associated with an increase in the number of severe exacerbations (p = 0.004), in the use of Long-term oxygen therapy (p = 0.006) and with reduced survival (p = 0.001). Similarly, a greater number of co-morbidities (≥ 3 co-morbidities) were associated with more severe systemic inflammation, more frequent use of mechanical ventilation or non-invasive ventilation (p=0.04) and reduced survival (p = 0.05).

**Conclusion:**

the presence of co-morbidities in patients with COPD is associated with higher severity and poorer prognosis.

## Introduction

La broncho-pneumopathie chronique obstructive (BPCO) est une maladie fréquente. Elle constitue un problème majeur de santé publique qu’on peut prévenir et traiter [1]. Elle se caractérise par des symptômes respiratoires persistants et une limitation chronique des débits aériens secondaire à des anomalies des voies aériennes et/ou des anomalies alvéolaires causées par une exposition importante à des particules ou des gaz nocifs [1]. Cette pathologie est émaillée d’une lourde morbidité et mortalité [1]. Elle constitue actuellement la quatrième cause de décès dans le monde et les projections prévoient qu’elle sera la troisième cause de décès en 2020 [1, 2].

Le diagnostic de la BPCO est spirométrique, il repose sur un rapport volume expiratoire maximal seconde (VEMS)/ capacité vitale forcée (CVF) < 70% après administration de bronchodilatateurs [1]. La BPCO est rarement isolée, elle est souvent associée à différentes comorbidités [1, 3]. Les mécanismes physiopathologiques expliquant la prévalence accrue des comorbidités dans la BPCO sont diverses. Certaines affections sont indépendantes de la BPCO, d’autres partagent avec celle-ci les mêmes facteurs de risque, majoritairement le tabagisme, d’autres résultent de la même physiopathologie, en occurrence l’inflammation systémique chronique, et certaines apparaissent comme une complication du traitement de la BPCO [1, 3-5].

Plusieurs études ont démontré que la majorité des patients atteints de BPCO était multi-tarée, cependant la multi-morbidité des patients ayant une BPCO demeure sous diagnostiquée et sous traitée et son impact sur l’évolution et la sévérité de la maladie restent mal élucidés [6-8]. Certaines études ont mis l’accent sur l’importance des comorbidités associées à la BPCO, telles que l’ensemble des pathologies cardiovasculaires, le diabète, la dénutrition, le cancer du poumon, la dépression, l’anxiété, l’ostéoporose et l’anémie [1, 3, 4, 6, 7].

Ces comorbidités sont un nouvel enjeu médical et thérapeutique dans la BPCO, en effet, elles semblent être associées à des symptômes plus sévères, des exacerbations plus fréquentes, un pronostic plus sombre et un coût de soins plus élevé [1, 6, 7]. Les recommandations du *Global initiative for chronic Obstructive Lung Disease* (GOLD) et de l’American Thoracic Society (ATS) donnent une place importante à la détection et au traitement des comorbidités pour permettre une meilleure évolution et une meilleure qualité de vie des patients ayant une BPCO [1, 9]. L’intérêt de notre travail est de déterminer l'impact des comorbidités sur la sévérité et l'évolution de la BPCO.

## Méthodes

**Type de l’étude:** il s’agit d’une étude rétrospective, monocentrique, analytique descriptive et comparative, portant sur les dossiers de patients porteurs de BPCO ayant consulté et/ou ayant été hospitalisés au service de Pneumologie au Centre Hospitalo-Universitaire Fattouma Bourguiba de Monastir durant la période allant de Janvier 2000 jusqu’à Décembre 2017.

### Population cible

**Critères d’inclusion:** les patients inclus dans cette étude sont les malades porteurs de BPCO selon la définition du Global initiative for chronic Obstructive Lung Disease (GOLD) 2017 qui associe: la présence d’une symptomatologie respiratoire chronique faite de toux, expectorations et dyspnée d’effort, et la présence d’un trouble ventilatoire obstructif (TVO) à la spirométrie non complètement réversible défini par un rapport volume expiratoire maximum à la première seconde (VEMS)/ Capacité vitale forcée (CVF) < 70% post bronchodilatation. Tous les patients inclus dans cette étude sont ceux ayant bénéficié d’un suivi régulier d’au moins 12 mois permettant d’apprécier le nombre et la sévérité des exacerbations. Une recherche systématique des comorbidités a été faite à la première consultation ou hospitalisation et une réévaluation des pathologies associées a été faite à chaque consultation.

**Critères de non inclusion:** sont exclus de cette étude les patients porteurs d’affections respiratoires chroniques comportant un trouble ventilatoire obstructif permanant et ne rentrant pas dans le cadre des BPCO : l’asthme bronchique dans sa forme chronique avec dyspnée continue; Les cas frontières entre asthme et BPCO (Asthma COPD Overlap (ACO)); Les bronchiolites chroniques de l’adulte; Certaines formes de bronchiectasies compliquées de trouble ventilatoire obstructif. Nous avons exclu également les patients présentant une symptomatologie de bronchite chronique mais n’ayant pas eu une spirométrie confirmant le TVO persistant.

**Paramètres étudiés:** les patients ont été évalués sur le plan démographique (âge, sexe, tabagisme, indice de masse corporelle (IMC)), clinique (la dyspnée selon l'échelle mMRC (modified Medical Research Council), les exacerbations aigues (EA)), biologique (numération formule sanguine (NFS), gazométrie artérielle, dosage de protéine C réactive (CRP)), thérapeutique (traitement médicamenteux, oxygénothérapie, ventilation mécanique invasive (VMI), ventilation non invasive (VNI)), et évolutif (nombre d’exacerbations, recours à la VNI, durée de l’hospitalisation, mortalité). Les exacerbations aigues sont classées en: EA légère (contrôlée par un renforcement de traitement β2 de courte durée d’action (CDA)), EA modérée (nécessitant un traitement par une corticothérapie systémique et/ou une antibiothérapie), et EA sévère (nécessitant une hospitalisation). Le délai entre les différentes consultations varie entre un et trois mois en fonction de la sévérité de la BPCO. Pour les exacerbations traitées dans les autres unités de soins (dispensaire local ou autres services hospitaliers), la sévérité et le traitement instauré pour ces différentes exacerbations sont recueillis à partir de l’interrogatoire du patient aux différentes consultations. Les données concernant la survie sont recueillies à partir des dossiers pour les patients décédés à l’hôpital. Les patients porteurs de cancer bronchique localement invasif ou métastatique sont exclus de l’étude de la survie du fait que la survie de ces patients est plutôt liée à l’évolution de la pathologie néoplasique. Pour les autres patients, les informations sur la survie sont recueillies par contact téléphonique.

**Comorbidités:** on a considéré comme comorbidité toute affection associée à la BPCO qu’elle soit découverte avant le diagnostic de la BPCO ou apparue lors du suivi. Les principales comorbidités retenues sont : l’hypertension artérielle systémique (HTA), les cardiopathies notamment ischémiques, la dénutrition, le syndrome d’apnées hypopnées obstructives du sommeil (SAHOS), le diabète, l’anémie, le cancer broncho-pulmonaire (CBP), l’insuffisance rénale chronique (IRnC), l’ostéoporose et les troubles anxieux et dépressifs.

**Répartition de la population:** les patients ont été répartis en deux groupes: Groupe G0: incluant tous les patients ayant une BPCO isolée. Groupe G1: incluant tous les patients ayant au moins une comorbidité. Nous avons comparé les différents paramètres de sévérité de la BPCO entre les deux groupes. Par la suite, nous avons divisé les patients du groupe G1 en deux sous-groupes: Groupe A: incluant les patients ayant une ou deux conditions comorbides associées à leur BPCO. Groupe B: incluant les patients ayant au moins trois comorbidités associées à la BPCO. Les différents paramètres de sévérité de la BPCO ont été comparés également entre les patients des groupes A et B.

**Analyse statistique:** les données ont été saisies et analysées grâce au logiciel SPSS version 20 (Statistical Package for the Social Sciences). Nous avons calculé les fréquences pour les variables qualitatives. Nous avons aussi calculé les moyennes, les médianes, l’écart type (ET) pour les variables quantitatives. Nous avons utilisé le test de Khi2 ou le test de Fisher pour comparer les variables qualitatives, le test de Student pour comparer les variables quantitatives. La probabilité de survie a été étudiée par la méthode de Kaplan-Meier avec des comparaisons effectuées par les tests de log Rank et Breslow. Le seuil de signification statistique a été fixé à 5%.

## Résultats

En tout 1152 patients porteurs de BPCO ont été retenus pour cette étude. L'âge moyen était de 66 ans avec une nette prédominance masculine (97,9%). Le nombre moyen d'EA /an était de 2,4±1,7. Le délai moyen de suivi de nos patients était de 4,3 ans (Tableau 1). Dans notre série, 911 patients (79%) avaient des comorbidités associées à la BPCO. La prévalence des comorbidités associées à la BPCO dans notre population: G0: absence de comorbidités; G1: au moins une comorbidité associée. En effet, 257 (22,3%) patients avaient une seule comorbidité, 335 (29,1%) avaient deux comorbidités, et 319 (27,7%) avaient trois comorbidités ou plus. Parmi les principales comorbidités associées nous retrouvons les comorbidités cardiovasculaires. Elles étaient présentes chez 298 patients soit 25,9%. Les autres comorbidités présentes sont citées dans le Tableau 2.

**Tableau 1:** principales caractéristiques de la population d’étude

	Nombre/ Moyenne	Fréquence
N Patients	1152	
Age	66±10	
Genre (M)	1128	97,9%
Tabagisme (sevré et non sevré)	1142	99%
Tabagisme (PA)	59±29	
Comorbiditiés ≥ 1	911	79%
VEMS (L)	1,26±0,53	
VEMS (%)	45±17	
VEMS / CVF (%)	58±9	
PaO2 (mmHg)	70±12	
PaCO2 (mmHg)	40±7	
N EA/an	2,4±1,7	
N H en Pneumologie /an	1,03±0,97	
IRC	525	45,6%
Suivi (an)	4,3±3,1	

N: Nombre, M: masculin, PA: paquets-années, VEMS: volume expiratoire maximum seconde, EA: exacerbation aigüe, H: hospitalisation, IRC: insuffisance respiratoire chronique

**Tableau 2:** les principales comorbidités associées à la BPCO

Comorbidités	Nombre	Pourcentage
HTA	267	23,2
Cardiopathie ischémique	256	22,2
HTP	123	10,7
Arythmie cardiaque	71	6,2
AVC	31	2,7
Dénutrition	154	13,4
SAHOS sévère	34	3
Diabète	178	15,4
Anémie	381	33
Cancer bronchique primitif	102	9
Insuffisance rénale chronique	22	2
Ostéoporose	76	6,6
Trouble anxio-dépressif	43	3,7

HTA: hypertension artérielle, HTP: hypertension pulmonaire, AVC: accident vasculaire cérébrale, SAHOS: syndrome d’apnée hypopnée obstructive du sommeil, SAHOS sévère: index d’apnée-hypopnée ≥30/h, Dénutrition: indice de masse corporelle <18.5 kg/m^2^

**Figure 1:**
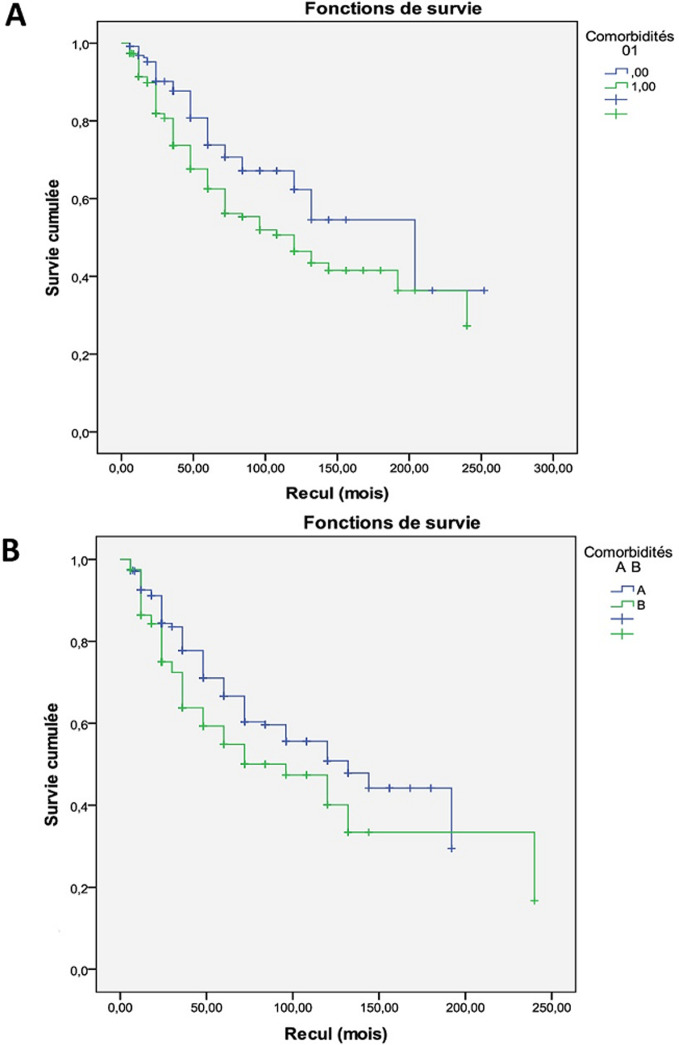
impact des comorbidités sur la survie des patients BPCO; (A) courbes de survie en fonction de la présence de comorbidités chez les patients BPCO; G0: absence de comorbidités; G1: au moins une comorbidité associée; (B) impact du nombre des comorbidités sur la survie des patients BPCO: GA: 1-2 comorbidités; GB: au moins trois comorbidités associées

Nous avons comparé d'abord deux groupes de patients: le groupe G0 comportant 241 (20,9%) patients n’ayant aucune comorbidité et le groupe G1 composé de 911 (78,9%) patients ayant au moins une comorbidité associée à leur BPCO. Le G1 était caractérisé par un nombre de femmes significativement plus élevé que le G0, avec un VEMS plus bas, une obstruction bronchique plus importante, un nombre d'hospitalisation pour EA plus élevé, et un recours plus fréquent à l'oxygène longue durée (OLD) (Tableau 3). La médiane de survie pour les patients indemnes de comorbidités (G0) était de 204 mois versus 120 mois pour les patients porteurs de comorbidités (G1) avec une différence statistiquement significative (p = 0,001). La survie pour les patients sans comorbidités à un an était de 97%, à deux ans de 95% et à cinq ans de 72%, alors que pour les patients ayant des comorbidités, la survie à un an était de 96%, à deux ans de 81% et à cinq ans de 62% (Figure 1 A).

**Tableau 3:** tableau comparatif entre les patients BPCO avec et sans comorbidités

	G0	G1	p
Age (ans)	64,7 ± 10,6	67,5 ± 10,1	0,12
Genre (M)	240 (99.5%)	888 (97.5%)	**0,04**
IMC (kg/m^2^)	22,9 ± 4,8	24,4 ± 5,6	0,08
Tabagisme (PA)	58,2 ± 31	60,1 ± 28,5	0,29
mMRC ≥2	123 (51%)	505 (55,4%)	0,1
VEMS (L)	1,29 ± 0,6	1,25 ± 0,5	**0,007**
VEMS/CVF post β2CDA (%)	59,1 ± 9,5	56,2 ± 10,2	**0,04**
pH	7,40 ± 0,04	7,40 ± 0,04	0,43
PaO2 (mmHg)	71,43 ± 12,12	69,74 ± 12,11	0,66
PaCO2 (mmHg)	40,08 ± 6,54	40,36 ± 6,88	0,58
Hémoglobine (g/dl)	12,9 ± 1,7	12,7 ± 1,8	0,2
CRP (mg/l)	72,8 ± 64	83,8 ± 77,6	0,15
N EA /an	2,3 ± 1,6	2,5 ± 1,6	0,8
N EA sévère/an	0,8 ± 0,6	0,9 ± 0,8	**0,004**
Durée H (jours)	8,67 ± 4,59	9,38 ± 4,94	0,38
N H en USI/patient/an	0,20	0,22	0,74
N VNI/patient/an	0,31	0,33	0,3
N VMI/patient/an	0,16	0,16	0,5
IRC	103 (42,7%)	422 (46,3%)	0,1
OLD	23 (9,5%)	147 (16,1%)	**0,006**

M: masculin, IMC: indice de masse corporelle, mMRC: modified Medical Resaerch Council, VEMS: volume expiratoire maximum seconde, CVF: capacité vitale forcée, β2CDA: bronchodilatateur β2 mimétique courte durée d’action, CRP: C reactive protein, N: nombre, EA: exacerbation aigüe, USI: unité de soins intensive, H: hospitalisation, VNI: ventilation non invasive, VMI: ventilation mécanique invasive, IRC: insuffisance respiratoire chronique, OLD: Oxygène longue durée

nsuite, on a divisé le groupe des patients ayant des comorbidités (G1) en deux sous-groupes: le groupe A (GA) comportant 592 patients (65%) ayant une ou deux comorbidités et le groupe B (GB) composé de 319 (35%) patients ayant au moins trois conditions comorbides associées à la BPCO. Le GB était caractérisé par un taux d'hémoglobine significativement plus bas, un taux de CRP plus élevé lors des EA sévères, avec plus de recours à la VNI et à la VMI (Tableau 4). La médiane de survie pour les patients ayant une ou deux comorbidités (groupe A) était de 132 mois versus 72 mois pour les patients ayant au moins trois comorbidités (groupe B) avec une différence statistiquement significative (p = 0,05). La survie pour le groupe A à un an était de 92%, à deux ans de 82% et à 5 ans de 64%, alors que pour le groupe B elle était de 88% à un an, de 78% à deux ans et de 56% à cinq ans (Figure 1 B).

**Tableau 4:** impact du nombre des comorbidités sur l’évolution de la BPCO

	GA	GB	p
Age (ans)	67 ± 10,5	68,6 ± 9,4	0,1
Genre (M)	575 (97,2%)	313 (98,1%)	0,25
IMC (kg/m^2^)	23,9 ± 5,5	25,5 ± 5,7	0,35
Tabagisme (PA)	57 ± 30	60 ± 29	0,23
mMRC ≥2	317 (53,5%)	188 (58,9%)	0,06
VEMS (L)	1,25 ± 0,5	1,26 ± 0,4	0,09
VEMS/CVF post β2CDA (%)	58,7 ± 9,6	59,9 ± 9,3	0,45
pH	7,40 ± 0,03	7,40 ± 0,04	0,39
PaO2 (mmHg)	72 ± 12,2	69, 4 ± 11,9	0,74
PaCO2 (mmHg)	40,1 ± 6,8	40 ± 6,9	0,5
Hémoglobine (g/dl)	12,9 ± 1,7	12,4 ± 1,8	0,009
CRP (mg/l)	76,1 ± 69	95,8 ± 87,5	0,002
N EA /an	2,4 ± 1,6	2,5 ± 1,6	0,34
N EA sévère /an	1 ± 0,9	0,8 ± 0,9	0,72
Durée H (jours)	9,31 ± 4,99	9,49 ± 4,93	0,73
N H en USI/patient/an	0,20	0,25	0,07
N VNI/patient/an	0,32	0,36	0,04
N VMI/patient/an	0,15	0,19	0,04
IRC	269 (45,6%)	153 (48%)	0,53
OLD	97 (16,4%)	50 (15,7%)	0,42

M: masculin, IMC: indice de masse corporelle, mMRC: modified Medical Research Council, VEMS: volume expiratoire maximum seconde, CVF: capacité vitale forcée, β2CDA: bronchodilatateur β2 mimétique courte durée d’action, CRP: C reactive protein, N: nombre, EA: exacerbation aigüe, USI: unité de soins intensive, H: hospitalisation, VNI: ventilation non invasive, VMI: ventilation mécanique invasive, IRC: insuffisance respiratoire chronique, OLD: Oxygène longue durée

## Discussion

Les patients atteints de BPCO ont souvent une maladie chronique associée du fait des facteurs de risque communs tels que le tabagisme, le vieillissement et la sédentarité, en association avec l’inflammation chronique systémique [1, 4, 10]. Notre travail avait pour objectif de déterminer l'impact des comorbidités associées à la BPCO sur le cours évolutif de la maladie. Nous avons retrouvé que les patients qui avaient au moins une comorbidité avaient une fonction respiratoire plus altérée que les patients sans comorbidités, avec plus d'hospitalisations pour EA et nécessité d'OLD. Nous avons aussi noté que plus le nombre de comorbidités augmente, plus l'inflammation systémique était plus importante, avec une tendance vers l'anémie, et survenue d'EA plus sévère (recours à la VNI et à la VMI). Dans notre travail, 79% des malades avaient au moins une comorbidité associée à la BPCO. Une seule comorbidité était trouvée dans 22,3% des cas, 29% des patients avaient 2 comorbidités et 27,6% avaient 3 comorbidités ou plus. Les études dans la littérature montrent que la prévalence des comorbidités varie de 50% à 97,7% avec une prévalence accrue de la multi-morbidité définie par le fait d'avoir deux maladies chroniques ou plus [11-16]. En effet un large essai clinique randomisé chez 5993 patients BPCO (essai UPLIFT) a montré que les comorbidités sont retrouvées chez 87% des malades [11]. De même l’étude de Westerik *et al*. qui a inclus 14603 patients a objectivé que 88% des patients avaient au moins une comorbidité et que 23,1% ont plus que cinq comorbidité [12]. Dans notre étude, la dyspnée s’accroit avec la présence des comorbidités et avec le nombre de celles-ci. De plus, une obstruction bronchique plus sévère a été objectivé chez les patients ayant des comorbidités (p=0,007). Ceci nous a permis de conclure que les comorbidités sont associées à une maladie plus sévère, ce qui est en concordance avec les données de la littérature [1, 15, 17]. Dans un autre sens, la *Lung Health Study* a constaté, chez les patients ayant une BPCO légère à modérée, qu’une réduction de 10% de la valeur prédite du VEMS est associée à une augmentation de 28% de la fréquence des événements coronariens fatals [10]. Même après la correction statistique pour les facteurs de risque cardiovasculaires classiques, tels que l’âge, le tabagisme ou encore le taux de cholestérol, l’obstruction des voies aériennes constitue un facteur de risque indépendant d’événements cardiovasculaires. D'autre part, la proportion de patients ayant plus que deux comorbidités était significativement plus élevée chez les patients symptomatiques (de stade B et D) comparativement aux patients de stade A et C (p < 0,0001) [17].

La BPCO entraine des modifications fréquentes de l’érythropoïèse [18]. Bien que la polyglobulie soit la réponse physiologique classique secondaire à l’hypoxémie, il est prouvé que l’anémie est aussi fréquente que la polyglobulie [19]. L’anémie aggrave le pronostic fonctionnel des malades BPCO en majorant la dyspnée et en limitant l’exercice, elle aggrave également la qualité de vie et augmente la mortalité [19-21]. Plusieurs études ont retrouvé une fréquence élevée d’anémie, allant de 8% jusqu’à 19,3% [13, 14, 22]. D’autres études ont démontré que la prévalence de l’anémie augmente avec la progression de la maladie, elle est de 17% chez des patients porteurs de BPCO modérée à sévère et de 23% chez des patients ayant une BPCO et qui sont hospitalisés [19, 23]. Les mécanismes incriminés sont l’inflammation systémique chronique associée, la dénutrition avec les carences en fer, en folates ou en vitamine B12 et l’insuffisance cardiaque associée [24]. Dans notre étude, la prévalence de l’anémie était plus élevée que celle de la littérature; elle se situait à 33% des cas. D'autre part, l'augmentation du nombre de comorbidités était significativement associée à la baisse du taux d'hémoglobine. Nous avons constaté aussi un taux plus élevé de CRP chez les patients BPCO avec trois ou plus de comorbidités. Cela témoigne de l'inflammation systémique attribuée à la BPCO et aux différentes pathologies associées.

Dans notre étude, les patients ayant des comorbidités faisaient plus d’exacerbations aiguës sévères (p = 0,004), de même, un recours plus fréquent à la VNI et à la VMI était observé chez les patients ayant trois comorbidités ou plus (p = 0,04). Nos résultats sont en concordance avec ceux de la littérature. En effet, selon Fettal *et al*. [13], le nombre des exacerbations chez les BPCO ayant des comorbidités était en moyenne de 4,7/an avec un nombre d’hospitalisations de 5,5/an. D’autres études ont démontré que le nombre de comorbidités augmente le risque d’exacerbations, le risque d’hospitalisations, la durée d’hospitalisation et la mortalité au cours ou au décours de celle-ci [12, 25, 26]. Selon Westerik, les patients ayant une comorbidité associée à la BPCO, quelle que soit sa nature, font fréquemment plus que deux exacerbations par an en comparaison avec ceux ayant une BPCO seule (8,2% versus 5,7%, p < 0,001) [12]. D’autre part, certaines études ont recherché l’effet du traitement de certaines comorbidités sur les exacerbations de BPCO. Il a été suggéré que les statines, un médicament fréquemment prescrit chez les patients atteints de coronaropathie, sont associées à une diminution du risque d’exacerbations de BPCO et à une diminution du risque d'intubation [27, 28]. Mais, il semble que ces médicaments ne réduisent le risque d’exacerbation de BPCO qu’en cas de coronaropathie associée [29]. Cela suggère que l’action protectrice peut être secondaire à la réduction de l'inflammation vasculaire et soutient en outre la théorie de l’implication de l'inflammation dans les exacerbations.

La présence de comorbidités aggrave l’évolution naturelle de la maladie, multiplie le risque de mortalité, prolonge la durée d’hospitalisation indépendamment d’une insuffisance respiratoire associée, augmente les coûts de santé et altère profondément la qualité de vie des patients [1, 30-34]. Selon Smith, un nombre important de comorbidités altère plus la qualité de vie que la baisse du VEMS [25]. Dans l’étude Eclipse [35], la mortalité est multipliée par deux si on a plus de trois morbidités. En cas d’insuffisance cardiaque le risque de mortalité est multiplié par neuf et il est multiplié par sept en cas de diabète. Dans l’étude TORCH [36], les principales causes de mortalité de la BPCO sont les atteintes cardiovasculaires et le cancer pulmonaire. L’association d’un SAHOS à la BPCO s’accompagne d’un risque accru d’hospitalisations pour exacerbation [37] et d’un risque accru de décès [38]. Dans notre étude, la survie était meilleure chez les patients sans comorbidités (p = 0,001) et chez les patients ayant un nombre moins élevé de comorbidités (p = 0,05). De même, il a été démontré que la BPCO aggrave le pronostic de chacune des comorbidités. En effet et selon Mascarenhas, la baisse de 10% du VEMS s’accompagne d’une augmentation du risque de mortalité de cause cardiovasculaire de 28%, indépendamment des autres facteurs de risque cardiovasculaires dont le tabac [39]. D’autre part, selon l’étude de Donaldson *et al*. le risque d’infarctus du myocarde augmente d’un rapport de 2,3 dans les 5 premiers jours d’une exacerbation [40].

Notre étude est caractérisée par la taille importante de l’échantillon, le délai de suivi intéressant, et la disponibilité de nombreuses données. D'autre part, il y a peu d’études dans la littérature qui se sont intéressés à l’influence du nombre des comorbidités dans la BPCO sur le pronostic de la maladie. Cependant, ce travail n'est pas sans limites. D'abord, il s’agit d’un travail rétrospectif, donc on ne peut pas maitriser toutes les données cliniques et para cliniques, ce qui peut être une insuffisance dans le recueil des données et de leurs interprétations ultérieures. En effet, La prévalence de plusieurs comorbidités étaient moindres que celle de la littérature, ceci est dû au fait que le recueil des données sur les comorbidités était basé sur les propos des patients, en l’absence d’un dossier médical informatisé unifié permettant le recueil des données de manière objective. D'autre part, on n’a pas étudié le rôle de chaque comorbidité à part dans le devenir des malades BPCO. L’idéal serait de réaliser des études prospectives prolongées prenant en compte une population large et hétérogène de patients BPCO permettant ainsi de palier à ces insuffisances. Un dépistage des comorbidités par des questionnaires appropriés et validés pourrait aider à mieux illustrer le profil des comorbidités et comprendre leur influence sur l’évolution et la survie.

## Conclusion

Les comorbidités ont un impact péjoratif sur l'évolution de la BPCO et compliquent la prise en charge de cette maladie. On est donc confronté au défi de trouver des moyens pratiques et appropriés pour dépister et traiter ces comorbidités. Il existe un intérêt croissant pour développer une approche globale et multidisciplinaire de la gestion de la multi-morbidité.

### Etat des connaissances actuelles sur le sujet

- La broncho-pneumopathie chronique obstructive est fréquemment associée à des comorbidités;
- Ces comorbidités ont un impact péjoratif sur le cours évolutif de la maladie.

### Contribution de notre étude à la connaissance

- L’augmentation du nombre de comorbidités au cours de la BPCO était associée dans notre travail à la réduction de la survie; ainsi bien que la BPCO soit liée à une mortalité élevée, la présence de comorbidités aggrave davantage le pronostic de la maladie: cela implique une prise en charge globale du patient BPCO non pas centrée seulement sur la BPCO mais aussi sur les pathologies associées aussi;
- La présence de comorbidités au cours de la BPCO était liée significativement à un nombre plus important d'hospitalisation; ainsi ces comorbidités ont un impact économique en terme de coût de la santé relative à la BPCO; l'intégration de ces comorbidités dans le plan d'action de la BPCO pourrait ainsi réduire les dépenses de santé;
- L'inflammation systémique était plus importante chez les patients BPCO avec comorbidités; bien que la BPCO est déjà connue responsable d'une inflammation systémique, la présence de pathologies associées intensifie cette inflammation témoignant de la complexité des mécanismes impliqués dans ces interactions.
